# Sustainable Wax Coatings Made from Pine Needle Extraction Waste for Nanopaper Hydrophobization

**DOI:** 10.3390/membranes12050537

**Published:** 2022-05-20

**Authors:** Sergejs Beluns, Oskars Platnieks, Jekaterina Sevcenko, Mara Jure, Gerda Gaidukova, Liga Grase, Sergejs Gaidukovs

**Affiliations:** 1 Institute of Polymer Materials, Faculty of Materials Science and Applied Chemistry, Riga Technical University, P. Valdena 3/7, LV-1048 Riga, Latvia; oskars.platnieks_1@rtu.lv (O.P.); gerda.gaidukova@rtu.lv (G.G.); sergejs.gaidukovs@rtu.lv (S.G.); 2 Institute of Technology of Organic Chemistry, Faculty of Materials Science and Applied Chemistry, Riga Technical University, P. Valdena 3/7, LV-1048 Riga, Latvia; hiacint89@inbox.lv (J.S.); mara.jure@rtu.lv (M.J.); 3 Institute of Materials and Surface Engineering, Faculty of Materials Science and Applied Chemistry, Riga Technical University, P. Valdena 3, LV-1048 Riga, Latvia; liga.grase@rtu.lv

**Keywords:** dip-coating, spray-coating, byproduct application, circular economy, green technology

## Abstract

We combine renewable and waste materials to produce hydrophobic membranes in the present work. Cellulose nanopaper prepared from paper waste was used as a structural component for the membrane. The pine wax was reclaimed from pine needle extraction waste and can be regarded as a byproduct. The dip-coating and spray-coating methods were comprehensively compared. In addition, the solubility of wax in different solvents is reported, and the concentration impact on coating quality is presented as the change in the contact angle value. The sensile drop method was used for wetting measurements. Spray-coating yielded the highest contact angle with an average of 114°, while dip-coating reached an average value of 107°. Scanning electron microscopy (SEM) was used for an in-depth comparison of surface morphology. It was observed that coating methods yield significantly different microstructures on the surface of cellulose fibers. The wax is characterized by nuclear magnetic resonance (NMR) spectroscopy and differential scanning calorimetry (DSC). Pine wax has a melting temperature of around 80 °C and excellent thermal stability in oxygen, with a degradation peak above 290 °C. Fourier transform infrared spectroscopy (FTIR) was used to identify characteristic groups of components and show the changes on coated nanopaper. Overall, the results of this work yield important insight into wax-coated cellulose nanopapers and a comparison of spray- and dip-coating methods. The prepared materials have a potential application as membranes and packaging materials.

## 1. Introduction

Global production must strive to use renewable feedstock and raw materials, design safe chemicals and products, and reduce our dependence on fossil resources. Agriculture and forestry have the potential to be significant sources of biobased chemicals and materials. While most products are used in the long term, the primary sources of waste and pollution are various packaging materials and single-use products [1]. An emerging material, cellulose nanopaper, is known for its dense structure and good mechanical and barrier properties [2]. However, cellulose is sensitive to moisture because of its hydrophilicity, limiting its application [3]. As a result, a lot of the research has been focused on making cellulosic materials more hydrophobic and improving their properties in humid environments [4].

Hydrophobization of cellulosic materials can be performed in various ways, e.g., coatings, lamination, and thermal and chemical treatments [4]. Lamination with polymers is extensively used, but this can significantly limit the disposal and recycling routes [5]. The use of chemical treatments has been widely explored for cellulose and offers a large variety of surface treatments. However, unfortunately, it requires chemicals and excess resources and can significantly increase the cost of production and produce toxic waste [6]. Coatings are highly adaptable in terms of materials, scalability, and method, i.e., spray- and dip-coating. What makes coatings especially attractive from a sustainability point of view is their relatively low resource usage, which affords competitive price solutions [7].

To preserve the biodegradability and non-toxicity of materials, various researchers have used natural waxes as coatings. Janesch et al. reported that a combination of tung oil and natural beeswax could be used to obtain superhydrophobic coatings on wood, with a static water contact angle above 150° [8]. Gupta et al. reported that carnauba wax from palm trees is superior to animal-based or mineral waxes and has exceptional de-wetting properties [9]. The coatings consisting of colloidal carnauba wax particles and poly-L-lysine have been suggested for cellulose nanofibril films and textiles, where authors showed that the combination of high hydrophobicity with good gas permeability could be achieved [10]. Other potential applications for natural wax involve various food and food packaging coatings [11,12]. Apicella et al. proposed using wax coatings for biodegradable plastic films to improve their surface hydrophobicity for applications in food packaging [13].

Pine trees are widely used to produce various wood and cellulose products, but often overlooked are the needles of these trees. In scientific literature, pine needles have been studied to monitor atmospheric pollution [14,15,16], while needle extracts are used in various medications and supplements [17,18]. Natural waxes are a byproduct of this extraction, often treated as waste. Thus, presenting the opportunity to examine pine needle integration into sustainable material production, and the fact that pine is one of the most common types of trees found worldwide, motivated this research. 

Nanopaper production can be carried out in a relatively clean route involving mechanical cellulose, wastepaper, and biowaste processing into nanofibrillated cellulose (NFC). Efficient mechanical processing of cellulose into nanocellulose has been demonstrated and is currently being tested in various industrial pilot plants [19,20]. This form of cellulose processing is only limited by electricity consumption, and avoids various toxic and corrosive processes [21,22]. NFC is a versatile material and can be used to prepare various membranes. Applications such as tight aqueous ultrafiltration membranes [23] and membranes for oil/water separation [24] have been proposed. Nevertheless, in nanopaper form, it can also be applied to food packaging, sensors, and even electronics [25,26].

In the present work, we developed hydrophobic coatings using pine needle wax, produced as a waste byproduct from the bioactive compound extraction process. For comparison, spray- and dip-coating methods were evaluated. Further, the influence of layer formation on the wetting and microstructure (SEM) was also investigated. The research data were supplemented with wax and nanopaper characterization using nuclear magnetic resonance (NMR) spectroscopy, differential scanning calorimetry (DSC), and Fourier transform infrared spectroscopy (FTIR). We demonstrate that pine wax-coated nanopaper membranes could provide a potential solution for developing moisture-resistant packaging materials.

## 2. Materials and Methods

### 2.1. Materials

Isopropyl alcohol was purchased from Honeywell (Hanover, Germany). Petroleum solvent, Nefras grade C2-80/120, was purchased from “Latvijas ķīmija” (Riga, Latvia). Activated charcoal powder, chloroform-d, Kraft lignin, and beechwood xylan were purchased from Merck KGaA (Darmstadt, Germany). Nanofibrillated cellulose (NFC) was produced from old filter paper waste according to the method previously reported by the authors [27]. NFC sizes ranged from 40 to 120 nm in diameter, and fiber lengths from around 500 to 2000 nm. Pine needles (*Pinus sylvestris*, L.) were gathered locally in Latvia through a forestry company. 

### 2.2. Fabrication of Nanopapers

First, 25 g of Kraft lignin was suspended in 235 mL of deionized (DI) water and magnetically stirred for 1 h at 85 °C. Using a robust alkaline solution (NaOH, Merck KGaA, Darmstadt, Germany), the suspension was fixed at pH 10. After excess water evaporation, a dark brown, homogenous suspension with a lignin content of 10 wt.% was obtained. Second, 25 g of beechwood xylan was dissolved in 240 mL of DI water and magnetically stirred for 1 h at 80–85 °C. After excess water evaporation, a brownish, homogenous, viscous solution with a xylan content of 10 wt.% was obtained. To prepare nanopapers, a 1 wt.% suspension of NFC in DI water was used.

NP (nanopaper) was cast in polystyrene Petri dishes (d = 146 mm). The NP cast from pure NFC suspension was abbreviated as CNP. In addition, modified NP was prepared with reduced hydrophilicity by mixing previously prepared lignin, xylan, and NFC suspensions via magnetic stirring for 2 h. The modified NP, abbreviated as MNP, was prepared with 98 wt.% NFC and 1 wt.% each xylan and lignin. 

### 2.3. Pine Needle Wax Extraction and Wax Characterization

**Figure 1:**
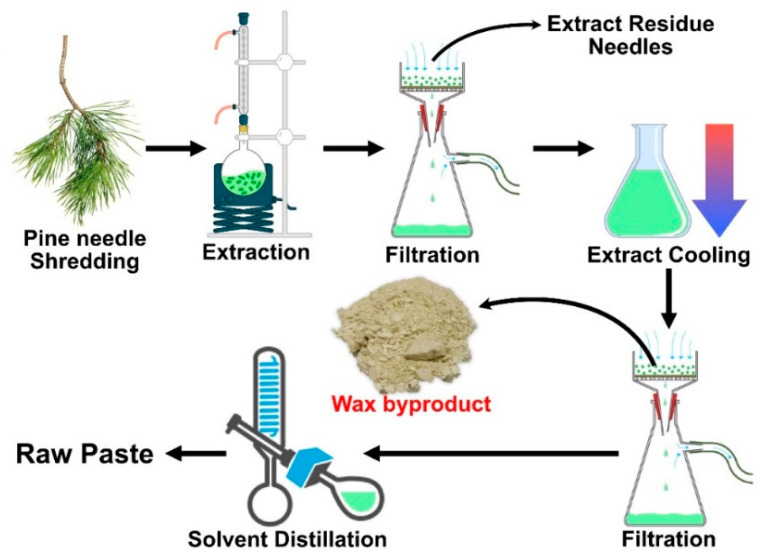
Schematic representation of pine needle extraction process.

Lab-scale extraction was performed to mimic industrial processing and is schematically presented in Figure 1. The Baltic pine (*Pinus sylvestris*, L.) needles were shredded with a blender (HENSKE 500 W; Vilnius, Lithuania). For 3 h, the needles were heated in hot Nefras solvent. The ratio of solvent to needles was 1:10 (*w/w*). The hot solution was filtered to separate the needles. The extracted solution was cooled and filtered again. This leaves a wax fraction in the filter, while valuable extracts remain in the solution. Wax was then purified by dissolving it in hot isopropyl alcohol (with a small amount of Nefras). Activated charcoal powder was added, and the hot solution was heated for 5 min, followed by filtration to remove charcoal agglomerates. The solution was left to cool down, and after the wax crystallized, it was filtered again. Yield: 2.35% (from needles); off-white solid (powder) with a slightly green shade. ^1^H NMR (300 MHz, CDCl_3_) δ 4.05 (t, *J* = 6.7 Hz, 2H, CH_2_–O–CO), 3.64 (t, *J* = 6.6 Hz, 1H, CH_2_–OH), 2.35 (d, *J* = 7.5 Hz, 2H, CH_2_–COO–H), 2.28 (t, *J* = 7.5 Hz, 2H, CH_2_–COO–CH_2_), 1.71–1.50 (m, 2H, CH_2_–CH_2_–O–CO–CH_2_–CH_2_–COO–CH_2_), 1.23 (s, *J* = 9.5 Hz, 11H, (CH_2_)_n_–CH_2_–CH_2_–O–CO–), 0.87 (t, *J* = 6.6 Hz, 1H, CH_3_–CH_2_). ^13^C NMR (75 MHz, CDCl_3_) δ 174.05, 64.42, 63.08, 34.41, 33.70, 32.79, 29.66, 29.61, 29.55, 29.51, 29.44, 29.27, 29.17, 28.65, 25.94, 25.74, 25.02, 24.74, 22.69, 14.13.

**Figure 2:**
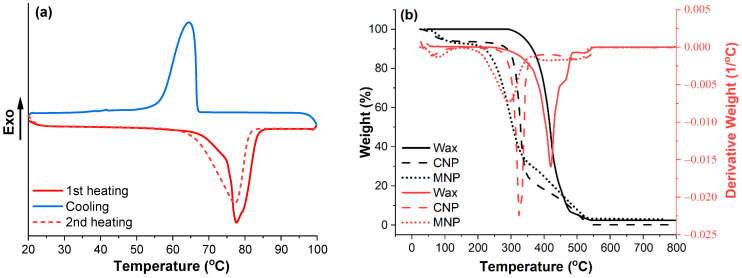
(**a**) Calorimetric properties of pine wax and (**b**) thermal stability of pine wax, CNP, and MNP.

The thermal properties of pine wax were measured with differential scanning calorimetry and are presented in Figure 2a. The heating curves show a difference between solution-crystalized (1st heating curve) and melt-crystallized (2nd heating curve) wax. The melting region (62–83 °C) of melted crystallized wax was shifted by about 3 °C to a lower temperature, but the melting peak was only 1 °C lower (77 °C). The crystallization process of wax started rapidly below the melting region and had a peak at 65 °C. The closest match for the obtained wax’s melting and crystallization parameters are those of candelilla wax [28,29]. Figure 2b shows the thermal stability of wax, and prepared cellulose NPs (CNP and MNP). The wax presented exceptional thermal stability in an oxygen environment, showing only a loss in mass starting at 295 °C and a maximum degradation temperature of 421 °C. The thermal stability curve of wax also revealed that it did not absorb any water, unlike CNP and MNP, which had about 6%. The overall thermal stability of wax exceeded that of CNP, but it cannot act as a protective layer for CNP or MNP due to wax melting.

### 2.4. Layer-by-Layer Fabrication of Pine Wax Coatings

**Figure 3:**
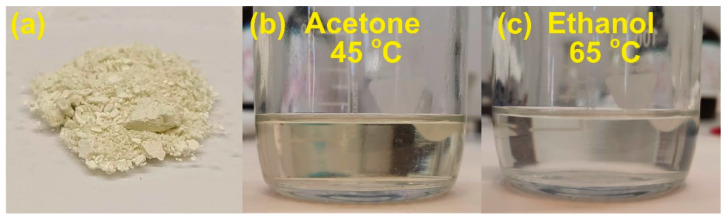
(**a**) Extracted pine wax powder and obtained solutions in (**b**) acetone and (**c**) ethanol.

Purified wax powder (Figure 3a) was dissolved in chloroform, hexane, ethanol, and acetone. The obtained results showed excellent solubility in chloroform, while other solvents required heating. Due to ethanol and acetone being less harmful solvents, we proceeded to examine them in heated solutions. The 1.0 wt.% of wax in acetone was fully soluble at 45 °C and in ethanol at 65 °C. The obtained solutions are shown in Figure 3b,c. As acetone performed better in lower temperatures, the rest of the research was conducted using acetone solutions.

Wax was dissolved in acetone to prepare 0.5, 1.0, and 1.5 wt.% solutions. Homogenization was achieved by 30 min of magnetic stirring and heating the solution at 45 °C. For spray-coating, the effective cone area was optimized, the distance from NP was set to 15 cm, and the spray solution temperature was kept between 43 and 47 °C. Three successive sprays were counted as a single-layer application (without pausing between them). Using 1.0 wt.% wax solution, three sprays (single layer) applied about 0.03 mg of wax coating on an area of 1 cm^2^. After spraying, the coated NP was inserted into the thermostat for 10 min at 60 °C. After 10 min in the thermostat, the next layer of spray-coating was applied in the same manner as described above.

The previously prepared 1.0 wt.% solution for dip-coating was kept between 43 to 47 °C. Prepared NP was submerged in the wax solution for 5 s. Using 1.0 wt.% wax solution, each dip (layer) applied about 0.05 mg of wax coating on the area of 1 cm^2^ (mass is given for a single side of NP for comparison to spray-coating). After dipping, the coated NP was inserted into the thermostat for 10 min at 60 °C. After 10 min in the thermostat, the next layer of dip-coating was applied in the same manner as described above.

### 2.5. Methods

^1^H and ^13^C{^1^H} NMR spectra were recorded using a Bruker 300 MHz (Billerica, MA, U.S.) spectrometer in CDCl_3_. Chemical shifts (δ) are reported in ppm and coupling constants (*J*) in Hz. Residual solvent (^1^H) or solvent (^13^C{^1^H}) peaks were used as an internal reference (for CDCl_3_ δ = 7.26 ppm and δ = 77.2 ppm).

Thermal testing was carried out on a Mettler Toledo DSC-1 (Greifensee, Switzerland), and calorimetric properties were measured in a nitrogen atmosphere for samples weighing an average of 10 mg. To obtain more precise results, the differential scanning calorimetry protocol included heating and cooling, followed by a second heating in the temperature range of 25 to 150 °C, with a heating/cooling rate of 10 °C/min, and samples were kept for 5 min at 150 and 25 °C.

Thermal stability was assessed using thermogravimetric analysis (TGA) on a Mettler TG50 (Greifensee, Switzerland) instrument. Data were measured on samples weighing about 10 mg. Heating was carried out in an oxygen atmosphere at a rate of 10 °C/min from 25 to 800 °C.

The structure of NPs was examined using an FEI Nova NanoSEM 650 Schottky field emission scanning electron microscope (FESEM, Hillsboro, OR, USA). Scanning electron microscopy (SEM) at a voltage of 10 kV was used to examine the morphology of nanopapers. Surface coatings were not applied.

The water contact angles of the NPs and the wax coating were measured using the static sessile drop method on a Theta Lite optical tensiometer (Attension^®^ Biolin Scientific, Gothenburg, Sweden). Five separate measurements were obtained in 10 s using a drop of water (2 μL) deposited on the surface of the specimen.

Fourier transform infrared spectroscopy (FTIR) in attenuated total reflectance mode was used to investigate NP and wax using a Nicolet 6700 (Thermo-Scientific, Waltham, MA, USA) with a resolution of 4 cm^−1^ in the 800–4000 cm^−1^ range. For each specimen, sixteen measurements were taken, and the average spectrum was provided.

## 3. Results and Discussion

### Coating of Nanopapers

**Figure 4:**
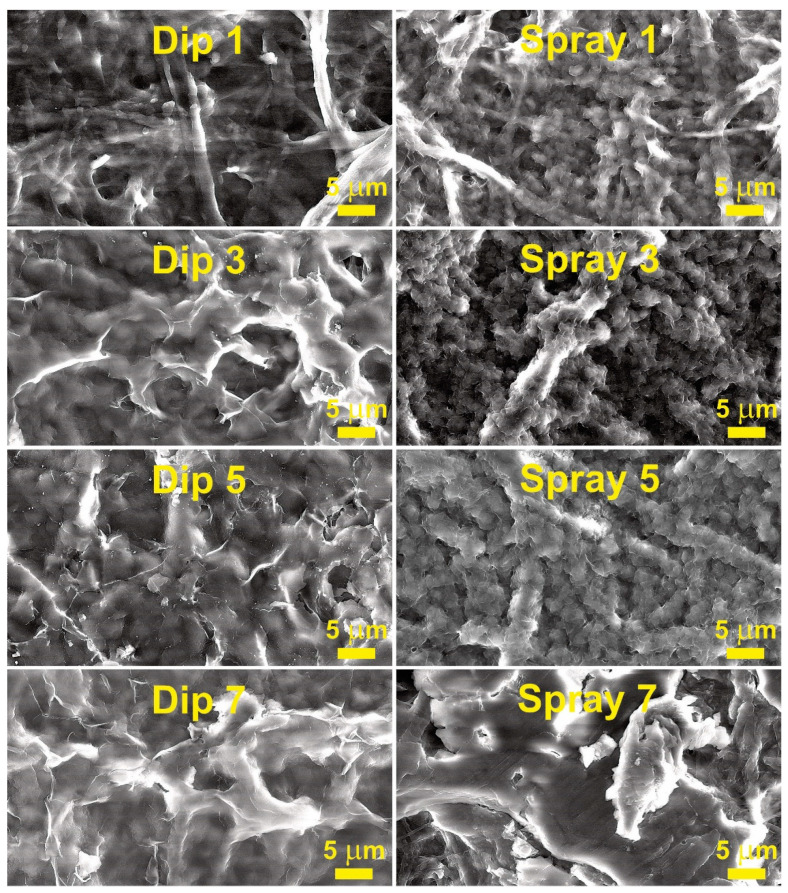
SEM micrographs of CNP surface with 1, 3, 5, and 7 layers of coatings applied by dip- or spray-coating methods using 1.0 wt.% wax solutions.

To understand the coating layer formation, SEM analysis was performed, and obtained micrographs are visible in Figure 4, and additional SEM micrographs are provided in Supplementary Figures S1 and S2. Both CNP and MNP have very similar microstructures, as indicated by SEM measurements (Figures S1 and S2); thus, wax layer formation was studied only for CNP. It is observable that both methods (spray and dip) yielded distinct microstructures. Spray-coating deposition produced the appearance of continuous spherical particle chains on the surface of cellulose fibers. In addition, all the spray samples at various layers demonstrated the presence of cracks (fractures) on the surface of coatings. However, dip-coating produced a relatively even and continuous coating on the surface of cellulose fibers. While some surface cracks were visible on dip-coatings, they were less frequent.

The second visible difference between coating methods was the amount of wax deposited, which matches the mass data from the methods section that showed that about 60% to 70% more wax is deposited by the dip method. Thus, the spray method visibly coats cellulose fibers, while the dip method, starting from three layers, forms a surface of the wax, where individual fibers are hard to distinguish.

Zhang et al. used bar-coating with a mixture of beeswax and carnauba wax (5:5) on paper and examined the influence of annealing temperatures [30]. The annealing temperature at 80 °C yielded similar SEM morphology to those obtained in this work, but lower temperatures yielded spherical particle layers on top of the paper. Wax coatings often deliver various shapes of morphologies as they are strongly influenced by the usage of solvent or emulsion and often come with annealing and post-annealing treatments [7]. In our case, we demonstrated that pine wax has good adsorption on cellulose fibers, but additional layers resulted in a separate wax layer above NP.

**Figure 5:**
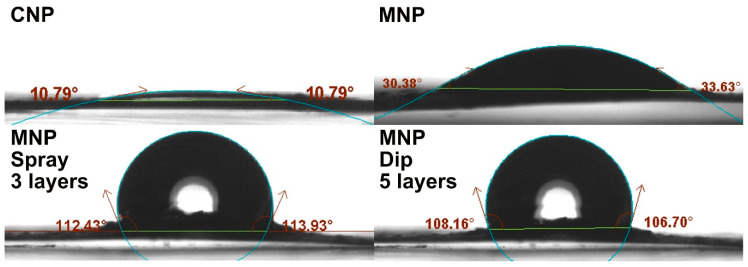
Contact angle measurements of CNP, MNP, spray-coated MNP with 3 layers of 1.0 wt.% solution, and dip-coated MNP with 5 layers of 1.0 wt.% solution.

**Figure 6:**
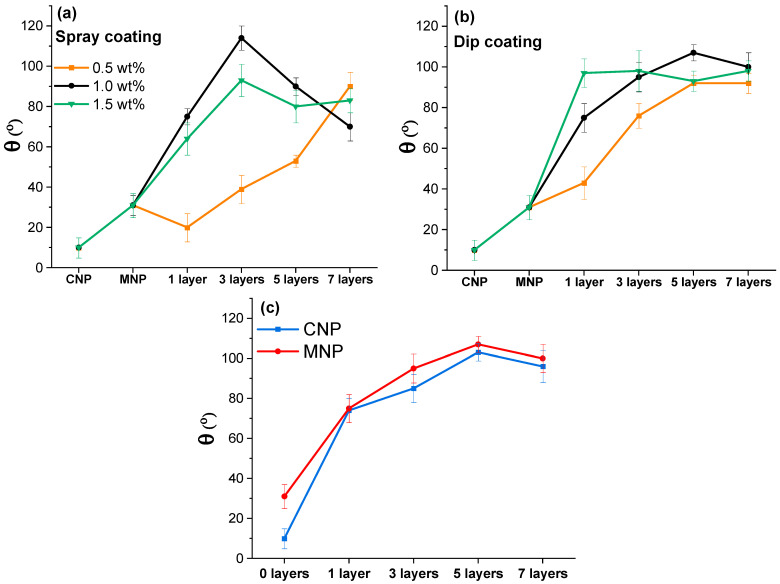
(**a**) Spray-coating and (**b**) dip-coating up to 7 layers of pine wax solution with different concentrations on the MNP. (**c**) Comparison of contact angle values for CNP and MNP using the dip-coating method with up to 7 layers of 1 wt.% pine wax solution.

Contact angles were measured for MNP and coated MNP with up to 7 layers to quantify the effect of wax deposition on hydrophobicity. The highest achieved contact angles on NP and coated NP are shown in Figure 5. Solution concentration can significantly impact the coating quality [31]. Concentrations of 0.5, 1.0, and 1.5 wt.% of wax solution were compared using the spray-coating method (Figure 6a) and the dip-coating method (Figure 6b). The curves show a significant difference in contact angle (θ) values, where the 0.5 wt.% concentration stands out with relatively low values. The 0.5 wt.% concentration yielded a gradual increase with each layer and had a maximum value at seven layers, reaching a contact angle of around 90° for both methods. At the same time, for spray-coated samples, the 1.0 wt.% solution yielded 75° after applying just one layer and a maximum value of 114° after three layers, showing an impressive increase of 44° and 83° over MNP. The additional layers showed a reversal in contact angle values and had a relatively steep decline. A higher concentration of 1.5 wt.% did not yield visible improvements for the spray method, showing lower values within the margin of error compared to 1.0 wt.% (except for three layers).

Dip-coating with 1.0 wt.% produces a more gradual increase than spray-coating, with the highest value of 107° after five layers and a slight drop to 100° after seven layers. The use of 1.5 wt.% yielded a very high value of 97° after just a single layer, but the successive layers did not show further improvement, and values remained around 95° to 100°. This indicates that the selected method and concentration significantly affect the formation of the coating after applying more than one layer, which matches the observations from SEM micrographs. With three coating layers, the spray method using 1.0 wt.% can achieve a hydrophobic surface (above 90°), while dip-coating requires just a single dip with a concentration of 1.5 wt.%. The relative change in coating performance is more consistent with dip-coated NPs, as there was a significant performance decrease by applying too many layers with the spray method. It has been reported that dip-coating is more efficient in industrial applications and forms highly uniform coatings, while spray-coating offers availability and simplicity [32].

**Figure 7:**
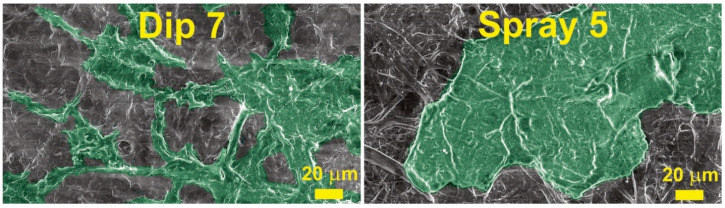
SEM micrographs with highlighted (green) wax agglomerates. The wax was applied with 1.0 wt.% solution.

To better understand the decrease in contact angle values after reaching a certain number of layers, colored SEM micrographs were prepared and are shown in Figure 7. As can be seen, a higher number of wax layers yielded agglomeration of wax particles on the surface of MNP. In this case, the agglomeration could be related to the solvent promoting the adhesion of wax particles that are subsequently drawn to large agglomerates and removed from some sections of fibers. In addition, it is known that surface roughness can significantly impact wettability [33,34]. Thus, initial layers promote a smooth coating of surface fibers, and a higher number of layers, i.e., above three for the spray method and above five for the dip method, results in much larger wax agglomerates on the surface of NP. The initial fibers visible in SEM (Figure 4) are from several hundred nm to 5 μm, but wax agglomerates after the application of seven layers are above sizes of 20 μm.

CNP and MNP samples were dip-coated to compare the nanopaper’s microstructure’s impact on the resulting contact angle values (Figure 6c). As demonstrated in our previous research, the pure CNP is more porous and has more hydroxyl groups on the surface compared to MNP [27]. The initial θ values showed that MNP and CNP are both highly hydrophilic, but MNP has an initial value of 31°, which is 3 times higher compared to CNP. Surprisingly, after just 1 layer of dip-coating, both NPs showed 74° and 75° contact angles, thus indicating that NP surface groups did not significantly influence the coating’s performance. With additional coating layers, CNP values showed a similar trend to those of MNP, with slightly lower values but within the error margin.

**Figure 8:**
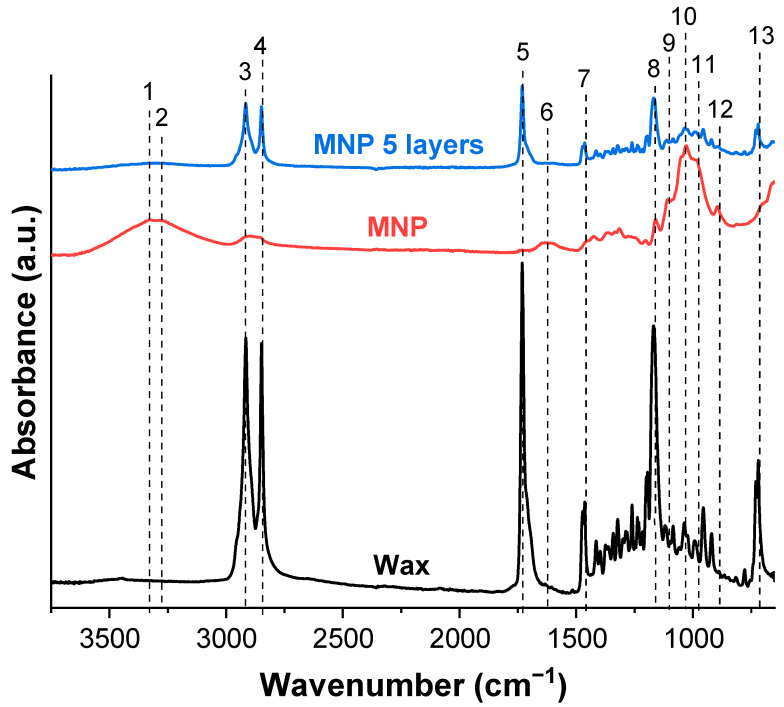
FTIR of pine wax, MNP, and five-layer dip-coated MNP with 1.0 wt.% wax solution (1–13 explained in Table 1).

FTIR analysis complements the previous methods by showing the changes in surface group chemistry for coated MNP. Figure 8 shows FTIR spectra of pine wax, MNP, and MNP with five layers of dip-coated wax, and Table 1 summarizes characteristic peaks. In this comparison, the most characteristic peak of cellulose was -OH group absorbance around 3300 cm^−1^. The distinct peaks of wax were visible at 2915 and 2849 cm^−1^, representing the stretching of CH_2_ groups [28,35], which matches well with cellulose CH and CH_2_ signals [27], but in terms of visual appearance are significantly different with the pronounced shape of the two peaks. Unique to wax is the signal at 1731 cm^−1^, which corresponds to the carbonyl group of the fatty acid ester linkage [28,35]. Absorbance at around 1600 cm^−1^ was attributed to -OH bending of absorbed water in cellulose or C = O stretching in the cellulose carboxyl group [36,37]. Like peaks around 2900 cm^−1^, the two peaks at about 1470 cm^−1^ are characteristic of wax but present in cellulose. Both can be attributed to C–H (CH_2_) bending, scissoring, and rocking vibrations [28,35]. The region from 1170 to 1000 cm^−1^ can be referred to as the fingerprint region, with a dense concentration of signals for both wax and cellulose, representing mainly various linkages of C-O. Absorbance at 976 cm^−1^ has been attributed to C-O stretching in xylan, 895 cm^−1^ to β-linkage of cellulose, and around 700 cm^−1^ to CH_2_ rocking vibrations [27,28,35]. After five layers of dip-coating, the coated MNP showed no -OH signals, and the overall shape of spectra matched the wax absorbance curve; thus, indicating successful hydrophobization of the NP surface.

## 4. Conclusions

In the last decade, wax-based coatings have gained attention as sustainable coatings for a variety of surfaces, including paper, wood, and metals. Pine wax characterization in the literature as a coating material is unexplored. However, pine is one of the most abundant trees in various climate conditions. In this research, we demonstrated a method of pine wax production during the pine needle extrication process. The pine wax can be considered a byproduct of this extraction, and the maximum yield achieved was 2.35%.

A solvent-based solution system was used to characterize the potential of pine wax coatings. The optimal coatings were achieved with three layers of spray-coating using 1.0 wt.% wax solution and one layer of dip-coating using 1.5 wt.% wax solution. The highest contact angle value of 114° was produced with three layers of spray-coating, while for the dip-coating, a slightly lower maximum of 107° (1.0 wt.% solution) was achieved with five layers. Using a 1.5 wt.% wax solution with the dip method resulted in the highest first-layer value of 97°. The addition of excess layers of wax resulted in a decrease in contact angle values. SEM micrographs showed significant changes in surface morphology with the increase of wax coating layers. FTIR analysis of coated nanopaper showed characteristic wax groups without the presence of hydrophilic cellulose hydroxyl groups. The thermal stability of pine wax showed exceptional stability in oxygen, but like other waxes, its relatively low melting point of 77 °C could be an issue in select applications.

The pine wax coating achieved a hydrophobic surface with a contact angle above 90°, but unfortunately, the values were not close to the superhydrophobic value of 150°. As we continue to seek more sustainable solutions and zero-waste technologies, pine needles provide an abundant source of renewable raw materials that are currently underutilized. The results will contribute to understanding the potential of natural waxes and the production of biodegradable hydrophobic surface coatings. In combination with dense nanopaper structures, the coatings produced have the potential to be used in a variety of food packaging applications.

## Figures and Tables

**Table 1:** Assignments of the infrared absorption bands.

Band	Wavenumber (cm^−1^)	Assignment	Reference
1	3325	Intramolecular hydrogen bonding of -OH group	[27]
2	3276	Intermolecular hydrogen bonding of -OH group	[27]
3; 4	2915, 2849	CH_2_ and C-H symmetrical and asymmetrical stretching	[27,28,35]
5	1731	C=O stretching vibrations of the monoester	[28,35]
6	1600–1640	-OH bending of absorbed water or C=O stretching in the cellulose carboxyl group	[36,37]
7	1472; 1464	C–H (CH_2_) bending, scissoring, and rocking vibrations	[28,35]
8	1168; 1160	C=O stretching vibrations of the monoester; C-H bending vibrations of the monoesters; C-O-C asymmetric bridge of cellulose	[27,28,35]
9; 10	1110, 1055, 1030	C-O stretching	[27,28,35]
11	976	C-O stretching in xylan	[27]
12	895	β-linkage of cellulose	[27]
13	700–720	CH_2_ rocking	[27,28,35]

## Data Availability

The data presented in this study are available on request from the corresponding author.

## Supplementary Materials

The following supporting information can be downloaded at: https://www.mdpi.com/article/10.3390/membranes12050537/s1, Figure S1: Lower-magnification SEM micrographs of the CNP surface with 0, 1, 3, 5, and 7 layers of coatings applied by the dip or spray method. Figure S2: Lower-magnification SEM micrograph of the MNP surface.
