# Supplementary material for: Sustainable Wax Coatings Made from Pine Needle Extraction Waste for Nanopaper Hydrophobization
Source: Membranes (Basel). 2022 May 20;12(5):537. doi: 10.3390/membranes12050537 (PMC9145576; doi:10.3390/membranes12050537)
Supplement: Supplementary file 1 [file membranes-12-00537-s001.zip › membranes-1714730-supplementary.pdf]

membranes

Supplementary Materials

Sustainable Wax Coatings Made from Pine Needle Extraction Waste for Nanopaper Hydrophobization

Sergejs Beluns [1,*], Oskars Platnieks [1], Jekaterina Sevcenko [2], Mara Jure [2], Gerda Gaidukova [1], Liga Grase [3] and Sergejs Gaidukovs [1]

[1] Institute of Polymer Materials, Faculty of Materials Science and Applied Chemistry, Riga Technical University, P.Valdena 3/7, LV-1048 Riga, Latvia; oskars.platnieks_1@rtu.lv (O.P.); gerda.gaidukova@rtu.lv (G.G.); sergejs.gaidukovs@rtu.lv (S.G.)
[2] Institute of Technology of Organic Chemistry, Faculty of Materials Science and Applied Chemistry, Riga Technical University, P.Valdena 3/7, LV-1048 Riga, Latvia; hiacint89@inbox.lv (J.S.); mara.jure@rtu.lv (M.J.)
[3] Institute of Materials and Surface Engineering, Faculty of Materials Science and Applied Chemistry, Riga Technical University, P.Valdena 3, LV-1048 Riga, Latvia; liga.grase@rtu.lv
* Correspondence: sergejs.beluns@rtu.lv

Citation: Beluns, S.; Platnieks, O.; Sevcenko, J.; Jure, M.; Gaidukova, G.; Grase, L.; Gaidukovs, S. Sustainable Wax Coatings Made from Pine Needle Extraction Waste for Nanopaper Hydrophobization. *Membranes* **2022**, *12*, 537. https://doi.org/10.3390/membranes12050537

Academic Editors: Cristiana Boi and Sanghyun Jeong

Received: 21 April 2022
Accepted: 18 May 2022
Published: 20 May 2022

Publisher's Note: MDPI stays neutral with regard to jurisdictional claims in published maps and institutional affiliations.

Copyright: © 2022 by the authors. Licensee MDPI, Basel, Switzerland. This article is an open access article distributed under the terms and conditions of the Creative Commons Attribution (CC BY) license (https://creativecommons.org/licenses/by/4.0/).

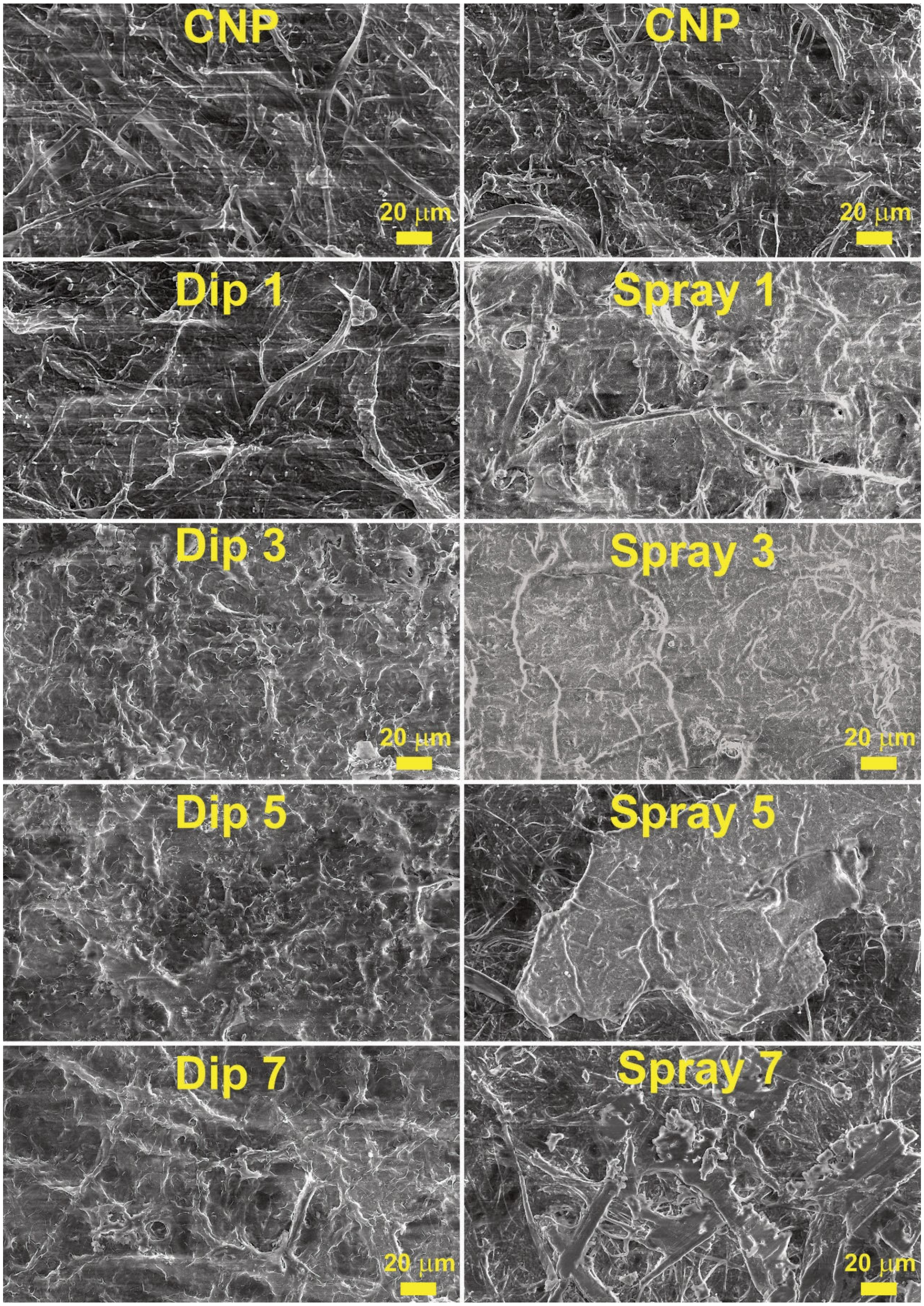

Figure S1. Lower magnification SEM micrographs of the CNP surface with 0, 1, 3, 5, and 7 layers of coatings applied by dip or spray method.

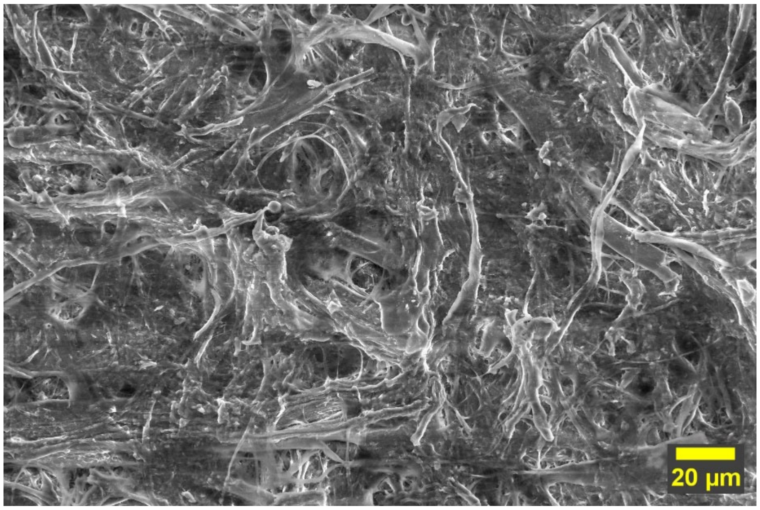

Figure S2. Lower magnification SEM micrograph of the MNP surface.
